# Crop pest image classification based on improved densely connected convolutional network

**DOI:** 10.3389/fpls.2023.1133060

**Published:** 2023-04-03

**Authors:** Hongxing Peng, Huiming Xu, Zongmei Gao, Zhiyan Zhou, Xingguo Tian, Qianting Deng, Huijun He, Chunlong Xian

**Affiliations:** ^1^ College of Mathematics and Informatics, South China Agricultural University, Guangzhou, China; ^2^ Key Laboratory of Smart Agricultural Technology in Tropical South China, Ministry of Agriculture and Rural Affairs, Guangzhou, China; ^3^ Center for Precision and Automated Agricultural Systems, Department of Biological Systems Engineering, Washington State University, Prosser, WA, United States; ^4^ College of Engineering, South China Agricultural University, Guangzhou, China; ^5^ College of Food Science, South China Agricultural University, Guangzhou, China; ^6^ Department of Asset and Laboratory Management, South China Agricultural University, Guangzhou, China; ^7^ College of Economics and Management, South China Agricultural University, Guangzhou, China

**Keywords:** pest image classification, selective kernel unit, representative batch normalization, DenseNet-121, ensemble learning

## Abstract

**Introduction:**

Crop pests have a great impact on the quality and yield of crops. The use of deep learning for the identification of crop pests is important for crop precise management.

**Methods:**

To address the lack of data set and poor classification accuracy in current pest research, a large-scale pest data set named HQIP102 is built and the pest identification model named MADN is proposed. There are some problems with the IP102 large crop pest dataset, such as some pest categories are wrong and pest subjects are missing from the images. In this study, the IP102 data set was carefully filtered to obtain the HQIP102 data set, which contains 47,393 images of 102 pest classes on eight crops. The MADN model improves the representation capability of DenseNet in three aspects. Firstly, the Selective Kernel unit is introduced into the DenseNet model, which can adaptively adjust the size of the receptive field according to the input and capture target objects of different sizes more effectively. Secondly, in order to make the features obey a stable distribution, the Representative Batch Normalization module is used in the DenseNet model. In addition, adaptive selection of whether to activate neurons can improve the performance of the network, for which the ACON activation function is used in the DenseNet model. Finally, the MADN model is constituted by ensemble learning.

**Results:**

Experimental results show that MADN achieved an accuracy and F1Score of 75.28% and 65.46% on the HQIP102 data set, an improvement of 5.17 percentage points and 5.20 percentage points compared to the pre-improvement DenseNet-121. Compared with ResNet-101, the accuracy and F1Score of MADN model improved by 10.48 percentage points and 10.56 percentage points, while the parameters size decreased by 35.37%. Deploying models to cloud servers with mobile application provides help in securing crop yield and quality.

## 1 Introduction

Agricultural pests have long posed a severe threat to the growth of crops and the storage of agricultural products (Cheng et al., 2017). The Food and Agriculture Organization (FAO) reported that these pests cause between 20 and 40 percent loss of global crop production every year. Because of relatively cheaper operational cost, farmers use a variety of chemicals such as pesticides to control pests, which has a negative impact on the agroecosystem (Geiger et al., 2010). If the location, time and listing of species and populations of invertebrate in the fields were available, instead of heavily relying upon pesticide, integrated pest management would use the optimized combination of mechanical, chemical, biological and genetic tools to mitigate harmful effects and enhance beneficial effects (Liu et al., 2016). Timely and accurate pest detection and classification are of great significance to its prevention and control, and early detection is a prerequisite to making an effective pest management plan and can reduce pollution.

Traditional crop pest classification relies mainly on manual observation or expert guidance, which is slow, inefficient, costly, and subjective. With the development of machine learning methods and computer vision techniques, researchers are beginning to use information technology to identify images of crop pests. The traditional machine learning classification framework consists of two main modules: the feature representation of the pest and the classifier. The normal used hand-crafted features include GIST (Oliva and Torralba, 2001), Scale Invariant Feature Transform (SIFT) (Lowe, 2004), Speeded Up Robust Feature (SURF), etc. The main classifiers commonly used include K-nearest neighbor classification algorithms (KNN), Support Vector Machines (SVM), etc. It is difficult to determine which of many features is optimal, and if the feature extraction is not correct, the subsequent classifier will make mistakes in identifying pests. With the advent of efficient learning algorithms for deep learning, it has achieved significant improvements in classification accuracy on many traditional classification tasks (Krizhevsky et al., 2017). In particular, convolutional neural networks (CNNs) are rapidly becoming the method of choice for overcoming certain challenges (Barbedo, 2018).

Recently, smart agriculture has been introduced to apply artificial intelligence (AI) technology, information and wireless communication technology applications. In addition, crop health monitoring is considered to be a major application of smart agriculture (Ayaz et al., 2019). Researchers are gradually turning their attention to designing mobile applications to identify pests. Karar et al. (2021) designed a mobile application using technologies such as Apache Cordova framework and Flask Web, and achieved good results in pest identification using deep learning techniques, but it used a relatively small dataset and identified only five categories of pests. Deep learning-based pest detection requires a large number of pest samples for supervised learning (Liu and Wang, 2021), and building an application that can identify multiple classes of pests in common crops is also in urgent need of development. It is well known that the ImageNet Large Scale Visual Classification Challenge (ILSVRC) (Deng et al., 2009) marks the beginning of the rapid development of deep learning, demonstrating that large-scale image data set play a key role in driving deep learning progress. However, most deep learning methods on insect pests are limited to small data set, and most public data set are collected indoor, which does not meet the needs of insect pest classification in field conditions. The IP102 large pest data set (Wu et al., 2019), which contains 75,222 images with a total of 102 classes from 8 crops, has alleviated this problem to some extent. However, the data set suffers from poor screening and misplaced pest categories, with a reported classification accuracy of only 49.4%. To address this issue, we invited agricultural experts and volunteers to further screen the IP102 data set. The new data set is of Higher Quality compared to IP102 and is named HQIP102.

The context of pest images in real environments is complex and suffers from large intra-class variation and small inter-class variation of pests. Existing models such as Densenet and ResNet do not work well on large pest datasets. To better identify larger pest data set, the DenseNet network (Huang et al., 2017), which performed well in the ImageNet task, is used as the base network. To improve the pest classification accuracy, we propose the MADN convolutional neural network model, which improves DenseNet-121 in three aspects: channel attention mechanism, input information feature enhancement and adaptive activation function. These improvements can improve the model’s pest classification performance.

The goal and objectives of our study are summarized as follows:

·Two criteria are used to further filter the IP102 large pest data set and improve the overall quality of the original data set, named HQIP102.
·Several techniques and the MADN convolutional neural network model are proposed to improve the representation capability of the DenseNet-121 network and improve its classification accuracy on large pest data set.

## 2 Related work

Research on crop pest classification based on computer vision has been a hot topic. In recent years, many computer-aided insect pest classification systems (Rani and Amsini, 2016; ; Alfarisy et al., 2018) are presented in the vision community. The methods involved mainly include machine learning and deep learning.

Machine learning often uses hand-crafted features such as SIFT, HOG (Dalal and Triggs, 2005), etc. Hand-crafted feature-based methods are the primary solutions for insect pest classification traditionally (Wu et al., 2019). Bisgin et al. (2018) used SVM to classify feature information such as size, color, basic pattern and texture extracted from 15 classes of food beetles, ultimately obtaining good classification results on a data set of 6900 images. Ebrahimi et al. (2017) designed an SVM structure with difference kernel function for thrips detection using the ratio of major diameter to minor diameter as region index as well as Hue, Saturation and Intensify as color indexes with a mean error of less than 2.25% for the best classification. Xiao et al. (2018) used SIFT image descriptor as well as SVM classifier to identify four important vegetable pests Whiteflies, Phyllotreta Striolata, Plutella Xylostella and Thrips with an average accuracy of 91.56% on 80 experimental images. Traditional machine learning algorithms rely on complex image processing techniques and handcrafted features, which often have limited robustness and generalization on large data set.

The successful application of deep learning in other fields has led to an increasing interest in agriculture, which is currently the most cutting-edge, modern, and promising technology (Kamilaris and Prenafeta-Boldú, 2018). Tetila et al. (2020) used transfer learning strategy to fine-tune Inception-v3, Resnet-50, VGG-16, VGG-19 and Xception to identify a data set containing 5000 soybean pest images. It has better performance compared to traditional feature extraction methods such as SIFT and SURF. Liu and Chahl (2021) used a novel approach to generate a virtual database that was successfully used to train a deep residual CNN with 97.8% accuracy in detecting four pests in agricultural environments. Khanramaki et al. (2021) proposed an ensemble classifier of deep convolutional neural networks to identify three common citrus pests with 99.04% accuracy on a data set containing 1774 images of citrus leaves. Ayan et al. (2020) used a weighted voting method to ensemble the pre-trained Inception-V3, Xception and MobileNet, which was named GAEnsemble, and its classification accuracy on the IP102 data set was 67.13%. Unlike Ayan et al. (2020), which used a fine-tuning strategy to combine existing models, this paper improves the DenseNet network and uses ensemble learning to combine the improved models.

Existing studies have shown that small datasets containing only a few pest classes have higher identification accuracy, while classification accuracy is low on the large data set IP102. To address the problem of misplacing pest categories in the IP102 data set, we built a Higher Quality pest data set named HQIP102. We also proposed the MADN convolutional neural network model for improving classification accuracy of existing models.

## 3 Materials and methods

### 3.1 Data set construction

Since IP102 contains more than 70,000 images of 102 categories, it inevitably has problems such as misplacement of some pest categories and lack of detailed screening.

**Figure 1:**
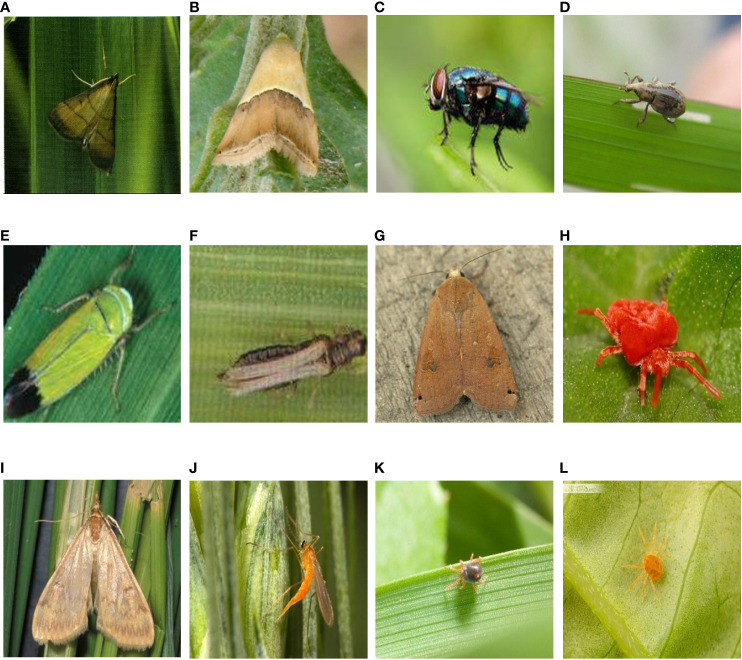
Sample images of some pests **(A)** rice leaf roller; **(B)** rice leaf caterpillar; **(C)** paddy stem maggot; **(D)** rice water weevil; **(E)** rice leafhopper; **(F)** grain spreader thrips; **(G)** yellow cutworm; **(H)** red spider; **(I)** corn borer; **(J)** wheat blossom midge; **(K)** penthaleus major; **(L)** longlegged spider mite;.

To obtain a higher quality pest data set, we invited agricultural experts and volunteers to further screen the IP102 data set according to the following two criteria. (1) obviously misplaced categories; (2) basically background, does not contain any target objects. The new data set is of higher quality and is named HQIP102. Low quality images are removed directly from the data set, Then the HQIP102 contains 102 pest categories for eight crops, including rice and wheat etc. Some of the pest image samples are shown in **Figure 1**.

As can be seen in **Figure 1**, the pest background in the HQIP102 data set is complex, the main part of the pest is small, and the similarity between some pest categories is high, which increases the overall classification difficulty. HQIP102 was filtered for each category of pests in IP102, with fewer images remaining for low quality pest categories, and the final HQIP102 pest data set contained 47,393 images. A comparison of HQIP102 with IP102 on eight crops corresponding to the pest category as well as the number of pests is shown in **Table 1**.

**Table 1:** Comparison of HQIP102 and IP102 on 8 crops.

Crop Category	Number of pest categories	IP102 Total	HQIP102 Total
Rice	14	8417	3006
Corn	13	14015	6373
Wheat	9	3418	2110
Beet	8	4420	1942
Alfalfa	13	10390	5611
Vitis	16	17551	14555
Citrus	19	7272	5173
Mango	10	9739	8623
Total	102	75222	47393

As can be seen from **Table 1**, HQIP102 filtered out more images on Rice, Corn, Beet, and Alfalfa, while fewer pest images were removed on the Wheat, Vitis, Citrus, and Mango categories. Among Rice crops, the rice leaf roller and asiatic rice borer categories have a higher number of deletions. In Corn crops, the corn borer and aphids categories removed more images. There are more images deleted from the beet army worm class in the Beet crop. In Alfalfa crops, alfalfa plant bug and blister beetle classes have more images deleted.

### 3.2 Data set split and dynamic data augmentation

The data set is divided into training set, validation set and test set according to the ratio of 7.5:1:1.5. The number of samples for certain pests in the data set is insufficient, and the use of data augmentation can increase the amount of data available for training, thus improving the generalization ability of the model. After splitting the data set, a dynamic data expansion method based on the number of pests in each class is proposed in this paper in order to solve the data imbalance problem in the HQIP102 training set, see Eq.1.


(1) $$N=\left\{ \begin{array}{l} 12N,0<N\leq 30\\ 7N,30<N\leq 60\\ 4N,60<N\leq 100\\ 3N,100<N\leq 150\\ 2N,150<N\leq 200 \end{array}\right.$$

Where N denotes the number of images in the training set for a particular type of pest. N is determined based on the average number of images of the pest category in the data set. The average number of images per pest category in the IP102 dataset is 460. And the specific pest image increase multiplier in the Eq.1 is adjusted manually, in which the range of the parameter *N* and the number of additional images are obtained by manual setting, to achieve the right amount of supplementary pest image data. With dynamic data augmentation, the data imbalance can be mitigated with a small amount of additional data, which is the basis for the parameter determination in Eq.1.

The data augmentation methods used were mainly a combination of center cropping, brightness contrast saturation adjustment, random horizontal flip, and random vertical flip. Specifically, the image is cropped to a size of 224 × 224 and has a 50% probability of random horizontal flipping and random vertical flipping. The probability of brightness and contrast adjustment is also 50%. The images are then saved to the original dataset after using data augmentation.

Using dynamic data enhancement, the total number of HQIP102 pest data set increased from 47,393 to 62,060 images, with the training set increasing from 35,607 to 50,274 and the validation and test sets remaining unchanged with 4734 and 7052. After using data augmentation, the ratio of training set, test set and validation set is about 8:1:1.

### 3.3 Dense convolutional network (DenseNet)

**Figure 2:**
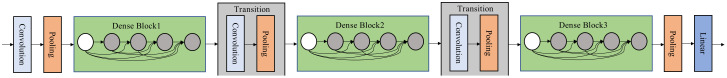
Structure of DenseNet with three dense blocks.

DenseNets (DenseNet-121, DenseNet-169, DenseNet-201, and DenseNet-264) alleviate the vanishing-gradient problem, strengthen feature propagation, encourage feature reuse, and reduces the number of parameters to some extent. In addition, the structure used by DenseNets shows good performance on large ImageNet datasets. For each layer, the feature-maps of all preceding layers are used as inputs, and its own feature maps are used as inputs into all subsequent layers. As shown in **Figure 2**, the network structure of DenseNet consists mainly of Dense Block and Transition.

In Dense Block, each layer has the same feature map size and can be concatenated in the channel dimension. All layers in the Dense Block output k feature maps after convolution, where the hyperparameter k is called the growth rate. We refer to each layer in a Dense Block as its substructure. Assuming that the number of channels in the feature map of the input layer is $k_{0}$ , then the number of channels in the input of layer l is $k_{0}+k\left(l-1\right)$ .

The Dense Block inside the DenseNet-B structure uses bottleneck layers to reduce the amount of computation. Transition layer, is mainly used to connect two adjacent Dense Blocks, and to reduce the size of the feature map. Its structure is Batch Normalization (BatchNorm) + ReLU + 1×1 Convolution + 2×2 AvgPooling. The Transition layer of the DenseNet-C structure also introduces a compression factor θ(<1) , which reduces the number of features in the output. When using bottleneck layers as well as transition layers with θ(<1) , such a model is called DenseNet-BC.

### 3.4 MADN convolutional neural network

**Figure 3:**
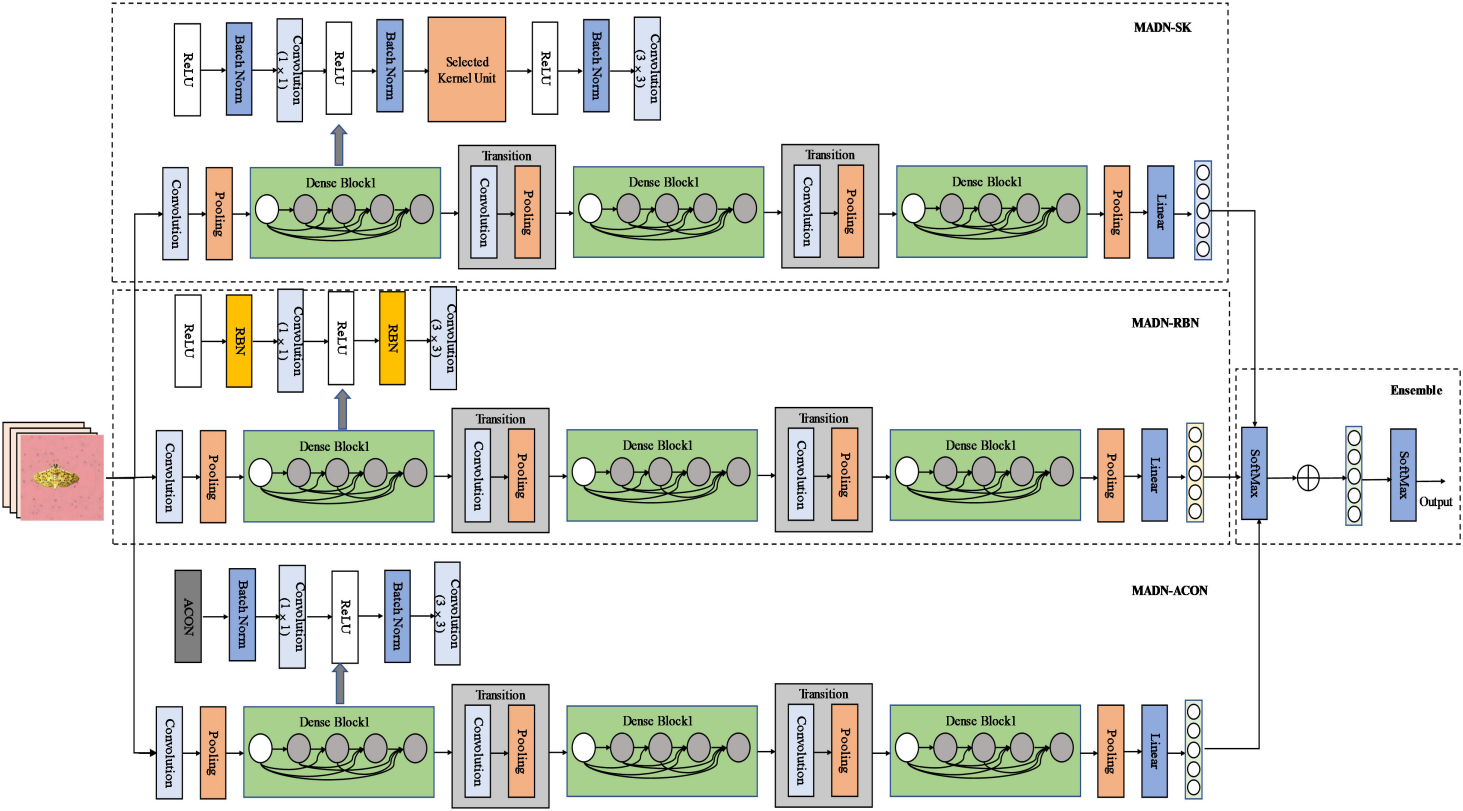
Structure of the MADN network model. The dense connection lines are omitted from the diagram, and the connections are made in the same way as the original DenseNet.

The MADN model focuses on improving the Dense Block structure in DenseNet in three ways, while the rest of the model is consistent with DenseNet. It introduces the Selective Kernel Unit (MADN-SK), the Representative Batch Normalization (MADN-RBN) module, and the ACON activation function (MADN-ACON) into the DenseNet. It is worth noting that MADN is not an end-to-end model, but combines 3 improved DenseNet models. Specifically, Using DenseNet-121 as the base network, MADN-SK, MADN-RBN and MADN-ACON are combined through ensemble learning to form the entire MADN model as shown in **Figure 3**. A detailed architectural comparison of DenseNet-121 with MADN-SK, MADN-RBN and MADN-ACON is shown in **Table 2**.

**Table 2:** Structural comparison of DenseNet-121 and modified models.

Layers	Output Size	DenseNet121	MADN_SK	MADN_RBN	MADN_ACON
Convolution	112×112	BN-ReLU-7×7 conv, stride 2			
Pooling	56×56	3×3 max pool, stride 2			
Dense Block(1)	56×56	BNReLU conv1; BN ReLu conv2 (6x)	ReLu BN conv1; ReLu BN SK; ReLu BN conv2 (6x)	ReLU RBN conv1; ReLu RBN conv2 (6x)	ACON BN conv1; ReLu BN conv2 (6x)
Transition Layer(1)	56×56	BN-ReLU-1×1 conv			
	28×28	2×2 average pool, stride 2			
Dense Block(2)	28×28	BN ReLU conv1; BN ReLu conv2 (12x)	ReLu BN conv1; ReLu BN SK; ReLu BN conv2 (12x)	ReLU RBN conv1; ReLu RBN conv2 (12x)	ACON BN conv1; ReLu BN conv2 (12x)
Transition Layer(2)	28×28	BN-ReLU-1×1 conv			
	14×14	2×2 average pool, stride 2			
Dense Block(3)	14×14	BN ReLU conv1; BN ReLu conv2 (24x)	ReLu BN conv1; ReLu BN SK; ReLu BN conv2 (24x)	ReLU RBN conv1; ReLu RBN conv2 (24x)	ACON BN conv1; ReLu BN conv2 (24x)
Transition Layer(3)	14×14	BN-ReLU-1×1 conv			
	7×7	2×2 average pool, stride 2			
Dense Block(4)	7×7	BN ReLU conv1; BN ReLu conv2 (16x)	ReLu BN conv1; ReLu BN SK; ReLu BN conv2 (16x)	ReLU RBN conv1; ReLu RBN conv2 (16x)	ACON BN conv1; ReLu BN conv2 (16x)
Classification Layer	1×1	7×7 global average pool			
		102D fully-connected, softmax			

where conv1 denotes a 1×1 convolution, and conv2 denotes a 3×3 convolution. MADN_SK, MADN_RBN, and MADN_ACON are the structures of the above modified DenseNet.

Sections 3.4.1 to 3.4.3 are the improvements of three aspects of DenseNet-121 in this study, each individual improvement is a complete model, and the final three models named MADN-SK, MADN-RBN, and MADN-ACON are obtained. Section 3.4.4 is an introduction to the ensemble learning used in this paper.

#### 3.4.1 MADN-SK

**Figure 4:**
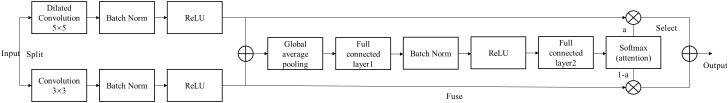
SK unit construction.

Li et al. (2019) proposes a dynamic selection mechanism in CNNs that allows each neuron to adaptively adjust its receptive field size based on multiple scales of input information. **Figure 4** shows the building blocks of the Selective Kernel (SK) unit.

In this building block, multiple branches with different kernel sizes are fused with softmax attention guided by information from these branches. The MADN-SK network is capable of adaptively adjusting the size of the receptive field according to the input to effectively capture target objects of different sizes, and its improved Dense Block substructure is shown in **Figure 4**.

#### 3.4.2 MADN-RBN

The BatchNorm module is widely used as it allows for more stable training of models. However, its centralization and scaling steps need to rely on the variance obtained from the sample statistics, ignoring the representation differences among instances. Gao et al. (2021) propose to add a simple yet effective feature calibration scheme into the centering and scaling operations of BatchNorm, namely Representative BatchNorm (RBN). The RBN is also divided into two steps: centering calibration and scaling calibration. For the entire process, see Eq.2.

Centering Calibration:


, $$X_{\text{cm}}=X+w_{m}K_{m}$$

Centering:


, $$X_{m}=X_{cm}-E\left(X_{cm}\right)$$

Scaling:


,(2) $$X_{s}=\frac{X_{m}}{\sqrt{Var(X_{cm})+\epsilon }}$$

Scaling Calibration:


, $$X_{cs}=X_{s}R(w_{v}K_{s}+w_{b})$$

Affine:


$$Y=X_{cs}\gamma +\beta$$

Where the input features $X\in R^{N\times C\times H\times W}$ , $w_{m}$ , $w_{v}$ , $w_{b}$ are the learnable weight vector. $K_{m}$ , $K_{s}$ represent the statistics of feature of each instance, which can be obtained using global average pooling. R() is a restriction function, often using sigmoid. E(X) and Var(X) denote the mean and variance and are used for centering and scaling. γ and β are learned scale and bias factors for affine transformation, and ϵ   is used to avoid zero variance.

The use of RBN to replace BN in DenseNet-121 allows better identification of crop pests, and experiments were conducted to verify this.

#### 3.4.3 MADN-ACON

Ma et al. (2021) propose a simple, effective, and general activation function ActivateOrNot (ACON), which learns to activate the neurons or not. ACON-C, see Eq. 3. ACON-C is one of the better-performing activation functions in ACON.


(3) $$\left(p_{1}-p_{2}\right)x\cdot \sigma \left(\beta \left(p_{1}-p_{2}\right)x\right)+p_{2}x$$

where β , $p_{1}$ and $p_{2}$ are learnable parameters and are channel-wise, the parameters are initialised randomly. We introduce ACON into the MADN model, which can improve the performance of the whole network.

#### 3.4.4 Ensemble learning

In the area of decision and risk analysis, information from several experts is aggregated by the decision maker, which can improve the accuracy of forecasts. For the ensemble of MADN-SK, MADN-RBN, MADN-ACON we considered the outputs of their classification layers, which determined the confidence values for each pest category. We used the sum of the normalized confidence values for each pest category on these three models as the final measure, see Eq.4.


(4) $$p^{'}i=\frac{\sum_{j=1}^{m}p_{ij}}{\sum_{i=1}^{n}\sum_{j=1}^{m}p_{ij}},i=1,\ldots ,n$$

Where $p_{ij}$ denotes the confidence value of the j-th network output for the i-th type of pest (in this paper m=3 , n=102 ). $p_{i}^{'}$ denotes the normalized value of the combined three network confidence values. The i-th pest label corresponding to the largest $p_{i}^{'}$ is chosen as the final prediction.

### 3.5 Experiment settings

To ensure fairness in the experimental comparisons, all experiments were built under the same conditions. The experiments were conducted on Ubuntu 18.04 with Intel(R) Core (TM) i9-10900K CPU and NVIDIA RTX3090 GPU with 24G memory. The RAM used is 32GB of DDR4, the deep learning tool is Pytorch 1.8, and the CUDA version is 11.4.The size of the input image was fixed at 224 ×224 and the optimizers were all used Adam (Adaptive momentum) (Kingma and Ba, 2014), the batch size was set to 64, the number of iterations was set to 50, and the learning rate was initialized to 0.001. The learning rate was reduced to half of the original rate if the model showed an increase in loss on the validation set during training.

### 3.6 Evaluation metrics

To better measure the classification performance of different models on the HQIP102 dataset, we chose Accuracy, Precision, Recall and F1Score as the evaluation metrics of the models.

Accuracy (Acc): The proportion of results predicted to be correct to the total sample, see Eq.5.


(5) $$A\text{cc}=\frac{TP+TN}{TP+TN+FP+FN}\times 100\%$$

Precision (Pre): The probability that all samples with a positive prediction are actually positive, see Eq.6.


(6) $$\mathrm{Pr}e=\frac{TP}{TP+FP}\times 100\%$$

Recall (Rec): The probability of all samples that are actually positive being predicted to be positive, see Eq.7.


(7) Rec=TPTP+FP×100%

F1Score (F1): The harmonic mean of precision and recall, see Eq.8.


(8) F1=2×Pre×RecPre+Rec×100%

In equations (5-7), TP indicates a true positive: the predicted is a positive sample and the actual is also a positive sample. TN indicates true negative: predicted negative sample, actual negative sample. FP indicates false positive: predicted positive sample, actual negative sample. FN indicates false negative: predicted negative sample, actual positive sample.

In addition, the model parameters, the GPU memory occupied during training, and the total training time were also used to measure the overall performance of the model. In particular, use the nvidia command in ubuntu to view the model’s GPU memory occupation, and the torch summary package in Pytorch to view the model’s parameters. Also, the inference time of each model for a single pest image is taken into account.

## 4 Results and discussion

### 4.1 Dynamic data augmentation experiments

On the training set of the original HQIP102 data set, we performed dynamic data augmentation based on the number of images of each type of pest. Using DenseNet-121 as the base network, the experimental results on the test set are shown in **Table 3**, keeping all factors consistent except for the different training data. As can be seen from **Table 3**, compared to the original data set, the DenseNet-121 network improved the accuracy by 0.41% and the F1 by 1.46%, the MADN network improved the accuracy by 1.15% and the F1 by 1.81%.Experiments show that the use of dynamic data augmentation techniques alleviates the problems caused by data imbalance to some extent with a small increase in the number of training samples.

**Table 3:** Dynamic data augmentation comparison experiments.

Data set	Method	Acc (%)	Pre (%)	Rec (%)	F1 (%)
HQIP102	DenseNet-121	70.11	61.43	58.96	59.66
HQIP102*	DenseNet-121	70.52	63.21	60.09	61.12
HQIP102	MADN	74.13	67.94	60.78	63.65
HQIP102*	MADN	**75.28**	**69.56**	**62.91**	**65.4**6

HQIP102* indicates the HQIP102 data set after using dynamic data augmentation. The bold values indicate the best values in this experiment.

### 4.2 Ablation experiments and comparative analysis

Ablation experiments were conducted to demonstrate the effectiveness of a series of improvements to the DenseNet-121 model. Accuracy and F1Score on the test set were used as metrics. The ablation experiments include the effect of using only SK units, RBN modules, ACON activation function and the final model after using ensemble learning. The Dense Block of DenseNet has been modified. When the SK unit is introduced, the model is named MADN-SK; when the RBN module is used, the model is named MADN-RBN, and when the ACON activation function is used to replace ReLU, the model is named MADN-ACON. Using ensemble learning to combine the advantages of the three modified models, the final model is named MADN. The results of the ablation experiments on the test set are shown in **Table 4**.

**Table 4:** Results of ablation experiments on the HQIP102 test set.

Model	Improvement method			Acc (%)	F1 (%)
	Selective Kernel unit	Representative BatchNorm	ACON activation		
DenseNet-121				70.52	61.12
MADN_SK	√			72.46	63.22
MADN_RBN		√		71.55	61.86
MADN_ACON			√	71.84	61.92
MADN	√	√	√	**75.28**	**65.46**

MADN is composed by ensemble learning. The bold values indicate the best values in this experiment.

**Figure 5:**
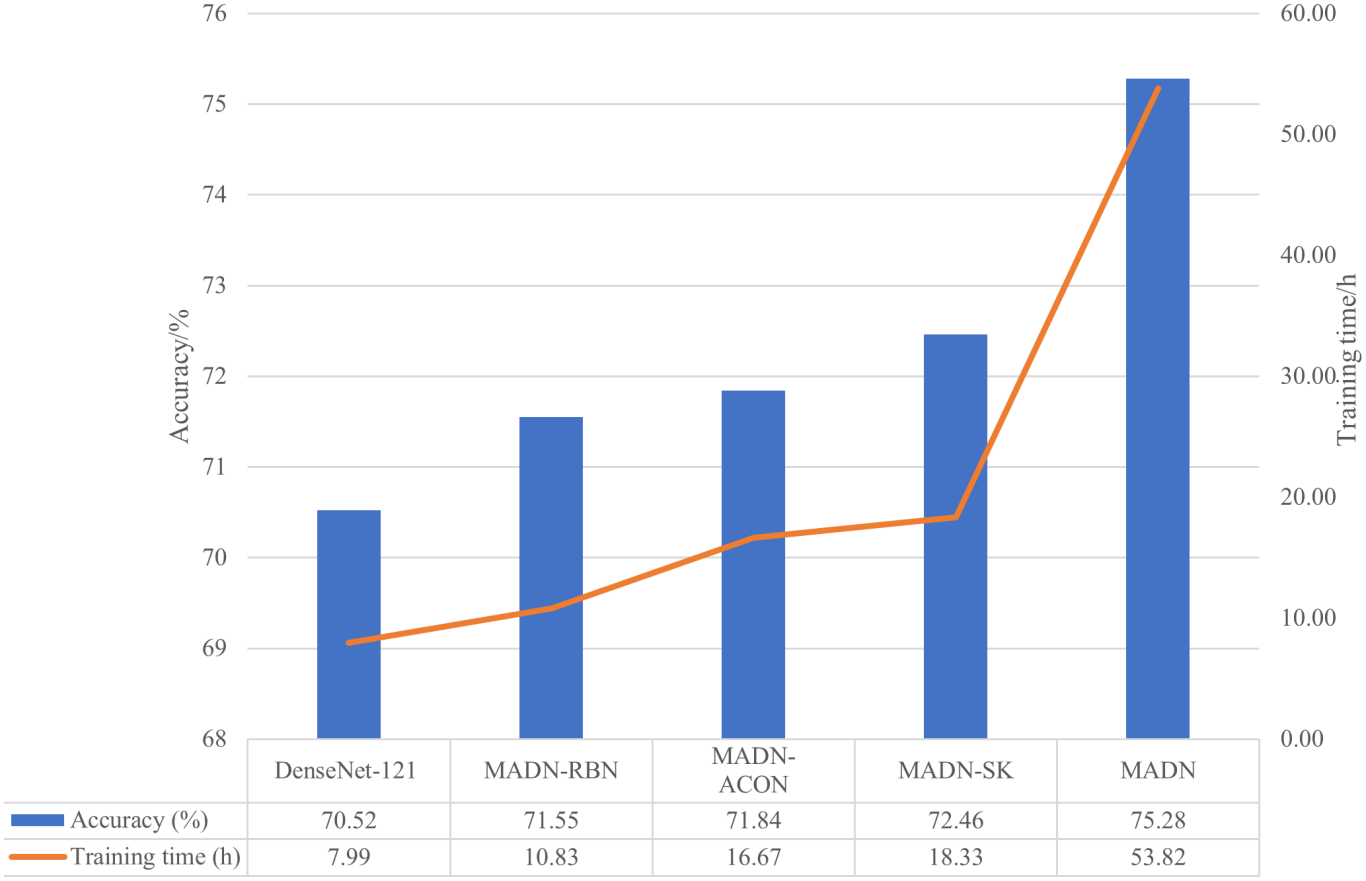
Comparison of training time and test set accuracy for DenseNet-121 and improved models.

As can be seen in **Table 4**, the improved MADN-SK, MADN-RBN, MADN-ACON and MADN all show better accuracy and F1Score compared to the DenseNet-121 model. MADN-SK obtained by introducing the Selective Kernel unit, which improved the accuracy on the test set by 1.94 percentage points and the F1Score by 2.1 percentage points compared to the pre-modified DenseNet-121;MADN-RBN, obtained using Representative BatchNorm, improved the accuracy and F1Score on the test set by 1.03 percentage points and 0.74 percentage points respectively; The MADN-ACON using the ACON activation function showed an accuracy improvement of 1.32 percentage points and an F1Score improvement of 0.8 percentage points on the test set. The MADN model using ensemble learning improved better, with accuracy and F1Score improvements of 4.76 and 4.34 percentage points respectively. As can be seen in **Figure 5**, During 50 iterations of training, the accuracy of the model gradually smoothed out on the validation set. And the improved MADN-SK, MADN-RBN and MADN-ACON have higher accuracy on the validation set compared to the original DenseNet-121 as the number of training iterations increases. From the experimental results in **Table 4**, it can be concluded that the improved MADN-SK, MADN-RBN, MADN-ACON and MADN are valid in improving the accuracy and F1Score compared to the origin DenseNet-121.

We compare the accuracy and training time of the DenseNet-121 as well as the improved classification model in **Figure 5**.

As can be seen in **Figure 5**, the improved MADN-RBN, MADN-ACON, and MADN-SK have improved accuracy on the test set at the expense of training time. MADN uses an ensemble learning strategy that requires pre-training of the MADN-RBN, MADN-ACON and MADN-SK models, so it requires more training time, but also higher accuracy on the test set. Although the training phase of a CNN model is usually time-consuming, it does not matter for the classification task, since the classifier is trained offline.

### 4.3 Comparison experiments with other models

**Figure 6:**
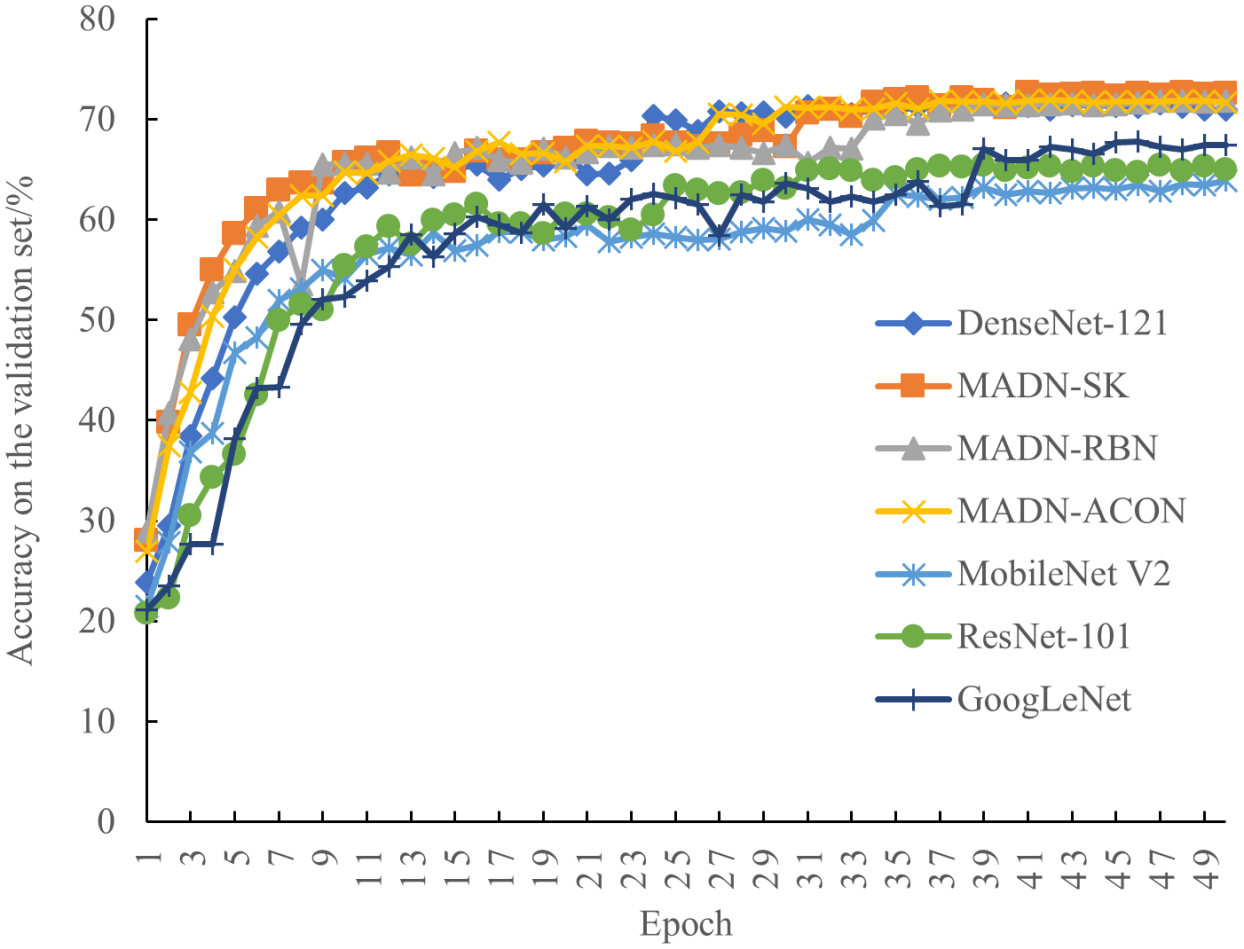
Classification accuracy of the model for each iteration on the validation set.

To better evaluate the performance of the improved MADN-SK, MADN-RBN, MADN-ACON, and MADN in this paper, accuracy, precision, recall, F1Score, GPU memory, training time, and parameters of the model were used as measures against ResNet-101 (He et al., 2016), GoogLeNet (Szegedy et al., 2015), MobileNet V2 (Sandler et al., 2018) for comparison experiments. The accuracy of each iteration on the validation set during training is shown in **Figure 6**, and the final experimental results on the test set are shown in **Table 5**.

**Table 5:** Performance of the model on the test set.

Model	Test set				Training phase		Parameters size (MB)	Inference time (ms)
	Acc (%)	Pre (%)	Rec (%)	F1(%)	GPU Memory (MB)	Training time(h)		
ResNet-101	64.8	56.88	54.19	54.9	11157	9.65	162.92	82.34
GoogLeNet	67.68	59.66	57.39	57.88	5687	2.85	21.76	17.67
MobileNet V2	63.63	55.65	53.79	54.25	6133	**2.44**	**8.98**	**13.41**
MADN	**75.28**	**69.56**	**62.91**	**65.46**	–	53.82	105.29	290.75

Since MADN is not an end-to-end network, it comes from combining 3 improved DenseNet networks by ensemble learning. Therefore, MADN cannot be trained alone, so “-” is used to indicate that the item does not exist. The bold values indicate the best values in this experiment.

As can be seen in **Figure 6**, the performance of each model on the validation set tends to stabilize as the iterations progress. Compared to the ResNet-101 and GoogLeNet models, MobileNet V2 performed relatively poorly. And compared to the other models, the improved MADN-SK, MADN-RBN and MADN-ACON show higher classification accuracy on the validation set.

As can be seen in **Table 5**, the lightweight model MobileNet V2 is optimal in terms of GPU capacity, training time and number of parameters, but performs poorly in terms of accuracy and F1Score on the test set; And compared to ResNet-101, GoogLeNet has a somewhat better overall performance; Although the improved MADN require more GPU memory and longer training time for training, they have better accuracy and F1Score compared to other models, and fewer number of parameters compared to the ResNet-101 model, which is more suitable for the practical needs of identifying pests and more suitable for deployment to cloud servers. Although the inference time of the MADN proposed in this paper is longer for a single pest image compared to other models, the application scenario of this study is to deploy the model to a cloud server, and the network transmission on the cloud server is inherently delayed, so the focus task of this study is to achieve better pest identification accuracy.

### 4.4 Experimental comparison of MADN and DenseNet-121 at the crop level

Considering the need for pest classification at the specific crop level, the test set accuracy of the improved MADN and DenseNet-121 models were compared on eight crops, as shown in **Table 6**.

**Table 6:** Experimental results of MADN and DenseNet-121 on eight crops test set.

Crop-Class	DenseNet-121	MADN
	Test set Acc	
Rice	59.68	**63.51**
Corn	70.54	**75.82**
Wheat	47.44	**53.53**
Beet	58.19	**64.11**
Alfalfa	61.08	**67.31**
Vitis	78.75	**82.66**
Citrus	68.54	**72.98**
Mango	75.37	**80.57**

The bold values indicate the best values in this experiment.

From **Table 6** we can see that the MADN network has improved accuracy for all eight crops, with classification accuracy exceeding 80% for both Vitis and Mango crops, an respective improvement of 3.91% and 5.2% compared to the pre-improvement DenseNet-121. Accuracy improvements were greater on Alfalfa and Wheat at 6.23% and 6.09% respectively. The accuracy of the model on different crops may be related to the size of the main part of the pest in different crops and the influence of background disturbances.

## 5 Conclusion

In this study, we filtered the IP102 data set and proposed a higher quality HQIP102 data set for pest classification, which includes 102 pest categories from eight crops with more than 40,000 images. To address the data imbalance, a dynamic data augmentation method is proposed, and the effectiveness of the method is experimentally demonstrated. The accuracy of the DenseNet-121 and MADN models on the HQIP102 dataset was improved by 0.41 and 1.15 percentage points, respectively, after using the data augmentation method. To resolve the issue of low classification accuracy of existing deep learning models on large pest data set, the DenseNet-121 was selected as the base network to be improved. In details, the DenseNet-121 was improved in three ways, i.e., MADN-SK, MADN-RBN and MADN-ACON networks. Also, such networks were combined to propose the MADN network. Validation experiments results showed the effectiveness of these improved methods was potential *via* increased accuracy, precision, recall and F1Score. Compared with the original DenseNet-121, the accuracy and F1Score of the MADN model on the HQIP102 dataset improved by 4.76 and 4.34 percentage points, respectively. We also carried out analysis at the crop species level, and experiments showed that the MADN network was more accurate for pest classification in Vitis and Mango, which could also be useful for related crop studies. Overall, the proposed deep networks will be helpful for crop pest precise management.

MADN is a combination of 3 improved DenseNet-121 models by ensemble learning, which cannot be trained end-to-end, and needs to train MADN-SK, MADN-ACON and MADN-RBN models first, so the consumption of inference time and training time are larger. In future work, we consider using end-to-end lightweight networks to reduce the training and inference time in scenarios with high requirements for recognition speed.

There are several possible reasons why MADN networks do not significantly improve prediction accuracy.

1. the HQIP102 dataset contains a large number of pest categories, and the similarity between different categories is large.

2. the background interference of pests is large, and the improved method can only improve the classification accuracy to a certain extent.

## Data availability statement

The original contributions presented in the study are included in the article/supplementary material. Further inquiries can be directed to the corresponding author.

## Author contributions

HP and CX designed research. HX and HH conducted experiments and data analysis. HX wrote the manuscript. ZG and ZZ revised the manuscript. XT and QD are responsible for contacting experts to filter the data set. All authors contributed to the article and approved the submitted version.
